# Rab43 GTPase directs postsynaptic trafficking and neuron-specific sorting of G protein–coupled receptors

**DOI:** 10.1016/j.jbc.2021.100517

**Published:** 2021-03-04

**Authors:** Zhe Wei, Xin Xu, Yinquan Fang, Mostafa Khater, Sean X. Naughton, Gang Hu, Alvin V. Terry, Guangyu Wu

**Affiliations:** 1 Department of Pharmacology and Toxicology, Medical College of Georgia, Augusta University, Augusta, Georgia, USA; 2 Department of Pharmacology, Nanjing Medical University, Nanjing, China

**Keywords:** G protein-coupled receptor, α_2_-adrenergic receptor, muscarinic acetylcholine receptor, Rab GTPase, Rab43, neuron, dendrite, synapse, trafficking, sorting, [^3^H]-NMS, [N-methyl-^3^H]-scopolamine methyl chloride, α_2_-AR, α_2_-adrenergic receptor, BFA, brefeldin A, BRET, bioluminescence resonance energy transfer, CFP, cyan fluorescent protein, ER, endoplasmic reticulum, ERK1/2, extracellular signal–regulated kinase 1 and 2, GPCR, G protein-coupled receptor, HEK293, human embryonic kidney 293, ICL3, third intracellular loop, M3-2B, M3R mutant containing the α_2B_-AR ICL3, M3R, M3 mAChR, mAChR, muscarinic acetylcholine receptor, Oxo-M, oxotremorine-m, sgRNA, single-guide RNA

## Abstract

G protein–coupled receptors (GPCRs) are important modulators of synaptic functions. A fundamental but poorly addressed question in neurobiology is how targeted GPCR trafficking is achieved. Rab GTPases are the master regulators of vesicle-mediated membrane trafficking, but their functions in the synaptic presentation of newly synthesized GPCRs are virtually unknown. Here, we investigate the role of Rab43, *via* dominant-negative inhibition and CRISPR–Cas9–mediated KO, in the export trafficking of α_2_-adrenergic receptor (α_2_-AR) and muscarinic acetylcholine receptor (mAChR) in primary neurons and cells. We demonstrate that Rab43 differentially regulates the overall surface expression of endogenous α_2_-AR and mAChR, as well as their signaling, in primary neurons. In parallel, Rab43 exerts distinct effects on the dendritic and postsynaptic transport of specific α_2B_-AR and M3 mAChR subtypes. More interestingly, the selective actions of Rab43 toward α_2B_-AR and M3 mAChR are neuronal cell specific and dictated by direct interaction. These data reveal novel, neuron-specific functions for Rab43 in the dendritic and postsynaptic targeting and sorting of GPCRs and imply multiple forward delivery routes for different GPCRs in neurons. Overall, this study provides important insights into regulatory mechanisms of GPCR anterograde traffic to the functional destination in neurons.

G protein–coupled receptors (GPCRs) constitute the largest superfamily of cell surface signaling proteins and modulate a variety of physiological and pathological functions of the nervous system. Neurons are the most sophisticated cells with specialized morphology and compartmentalization in which most GPCRs are expressed in both presynaptic and postsynaptic membrane terminals where they are able to bind to respective neurotransmitters to activate cognate heterotrimeric G proteins or other signaling molecules which in turn activate downstream effectors, including ion channels, and thus control synaptic signaling and transmission (1, 2, 3).

GPCRs are synthesized in the endoplasmic reticulum (ER). After being correctly folded and properly assembled, newly synthesized receptors can pass through the ER quality control system and export from the ER through the Golgi, along the axon and dendrites, to the synaptic compartments (4, 5). Although previous studies have demonstrated that GPCR surface transport in neurons is regulated by specific motifs and interacting proteins (6, 7, 8, 9, 10, 11), the molecular mechanisms underlying the synaptic targeting and sorting of nascent GPCRs are poorly defined.

Rab GTPases are the master regulators of vesicle-mediated membrane traffic in exocytic and endocytic pathways; they regulate a number of events involved in the development of central nervous system and neuronal functioning, such as neurite morphogenesis and outgrowth, axonal and dendritic transport, synaptic vesicle fusion and elimination, neurotransmitter release, and synaptic transmission (12, 13, 14, 15, 16, 17). Among more than 60 Rabs identified in mammals, some members are specific or enriched in neurons and perform neuron-specific functions, and ubiquitously expressed Rabs may have specialized functions in neurons (18, 19). For instance, Rab3 and Rab27, two best-characterized neuron-specific Rabs, control synaptic vesicle exocytosis and the efficiency of neurotransmitter release (20, 21, 22, 23, 24). Several Rabs have been shown to mediate agonist-induced GPCR endocytosis and the recycling of internalized receptors in neurons (25, 26, 27). However, virtually nothing is known about the function and regulation of the Rab family in the transport of newly synthesized GPCRs to dendrites and synapses.

To address these issues, in this study, we determine the role of Rab43 in the dendritic and synaptic transport of the prototypic family A α_2_-adrenergic receptor (α_2_-AR) and muscarinic acetylcholine receptor (mAChR). The α_2_-AR and mAChR have three (α_2A_-, α_2B_-, and α_2C_-AR) and five subtypes (M1R–M5R), respectively, and all play important roles in the central and peripheral nervous systems. As compared with many other secretory Rab GTPases, the function of Rab43 is relatively poorly defined. Previous studies have shown that Rab43 is important for Golgi structure (28, 29), ER–Golgi transport (30, 31), retrograde surface–Golgi transport (28), phagosome maturation (32), and antigen cross-presentation by dendritic cells (33). Here, we demonstrate that Rab43 mediates the dendritic and postsynaptic delivery of some but not all GPCRs, and the function of Rab43 in the sorting of different GPCRs is dictated by direct interaction and in a neuronal cell–specific manner. Our data also provide direct evidence implicating distinct biosynthetic pathways that deliver different GPCRs to the functional destinations in neurons.

## Results

### Rab43 selectively regulates the surface expression and signaling of endogenous α_2_-AR, but not mAChR, in primary neurons

**Figure 1:**
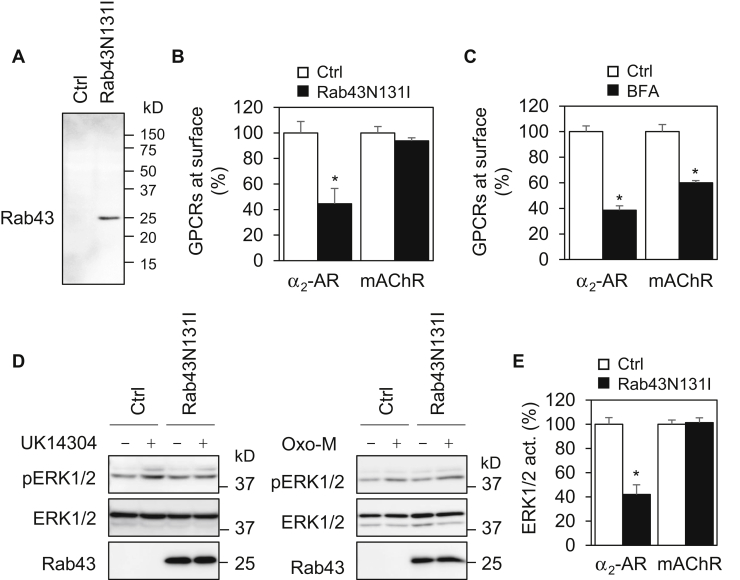
**Rab43 differentially regulates the surface expression and signaling of endogenous α**_**2**_**-AR and mAChR in primary neurons.***A*, lentivirus-mediated expression of Rab43N131I. Cortical neurons were cultured on 12-well dishes and infected with control or Rab43N131I lentiviruses for 48 h. Rab43 expression was measured by Western blotting using antibodies against human Rab43, which do not recognize rat Rab43. *B*, Rab43N131I inhibits the surface expression of α_2_-AR, but not mAChR. Cortical neurons were cultured and infected as above, and the surface expression of endogenous α_2_-AR and mAChR was determined by intact cell ligand binding using [^3^H]-RX821002 and [^3^H]-NMS, respectively, at 2 nM. In a typical experiment, the mean values of specific ligand binding of α_2_-AR and mAChR were 1425 and 3955 DPM, respectively, in neurons infected with control viruses. *C*, BFA treatment attenuates the surface expression of both α_2_-AR and mAChR. Cortical neurons were treated with dimethyl sulfoxide or BFA at 1 μg/ml for 24 h, and the surface receptor expression was measured by radioligand binding. *D*, Rab43N131I inhibits ERK1/2 activation by the α_2_-AR agonist UK14304, but not the mAChR agonist Oxo-M. Cortical neurons were cultured and infected as above. After starvation for 48 h, the neurons were stimulated with UK14304 at 1 μM or Oxo-M at 10 μM for 5 min. ERK1/2 activation was determined by measuring their phosphorylation by Western blotting. *E*, quantitative data shown in panel *D*. The quantitative data shown are the percentages of the mean value obtained from control neurons and are presented as the mean ± SD of at least three experiments. ∗*p* < 0.05 relative to respective control. [^3^H]-NMS, [N-methyl-^3^H]-scopolamine methyl chloride; α_2_-AR, α_2_-adrenergic receptor; BFA, brefeldin A; ERK1/2, extracellular signal–regulated kinase 1 and 2; mAChR, muscarinic acetylcholine receptor; Oxo-M, oxotremorine-m.

As an initial approach to study the possible function of Rab43 in regulating the anterograde transport of newly synthesized GPCRs in neurons, we measured the effect of lentiviral expression of dominant-negative guanine nucleotide-deficient mutant Rab43N131I on the surface expression of endogenous α_2_-AR and mAChR in primary cortical neurons in radioligand binding assays. Expression of Rab43N131I markedly reduced the surface number of α_2_-AR by approximately 55% as compared with neurons infected with control viruses (Fig. 1, *A* and *B*). Surprisingly, Rab43N131I expression had no significant effect on the surface expression of mAChR (Fig. 1*B*). In contrast, treatment with brefeldin A (BFA), which disrupts the Golgi structure and thus blocks the ER-to-Golgi transport, similarly inhibited the surface expression of both α_2_-AR and mAChR by 40 to 60% (Fig. 1*C*).

To define if Rab43 could differentially affect the concomitant function of α_2_-AR and mAChR, we used the activation of mitogen-activated protein kinases, specifically extracellular signal–regulated kinase 1 and 2 (ERK1/2), as a readout. Although α_2_-AR and mAChR each have multiple subtypes which couple to distinct heterotrimeric G proteins, they are all able to activate ERK1/2. Consistent with its effects on the surface receptor expression measured in radioligand binding assays, lentiviral expression of Rab43N131I strongly inhibited ERK1/2 activation in response to stimulation with the α_2_-AR agonist UK14304, without altering ERK1/2 activation by the mAChR agonist oxotremorine-m (Oxo-M) (Fig. 1, *D* and *E*). These data demonstrate that the surface expression of α_2_-AR and mAChR, as well as their signaling, is differentially regulated by Rab43 in neurons.

### Rab43 controls the dendritic and postsynaptic delivery of α_2B_-AR, but not M3R

**Figure 2:**
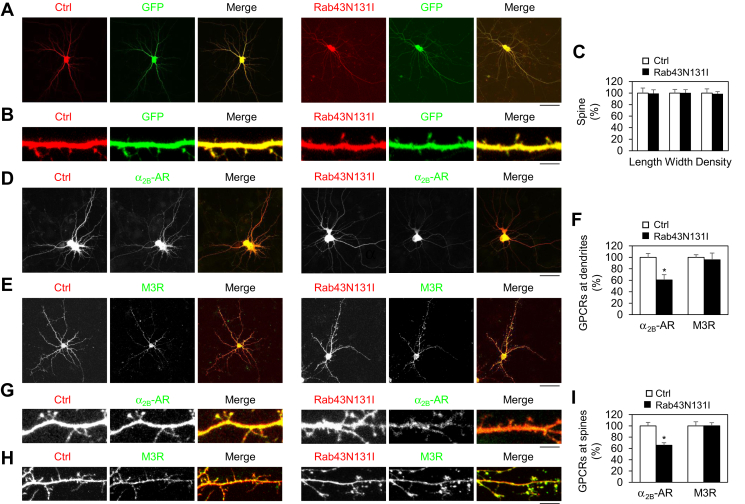
**Rab43N131I selectively inhibits the dendritic and postsynaptic delivery of α**_**2B**_**-AR, but not M3R, in hippocampal neurons.***A*, effects of Rab43N131I on neuronal morphology. The neurons were cultured on 12-well dishes and transfected with 250 ng of pEGFP-N1 vectors and 1250 ng of DsRed-C1 vectors or DsRed-Rab43N131I for 48 h. *B*, effects of Rab43N131I on spine morphology. *C*, effects of Rab43 on the dendritic spine length, width, and density as quantified by measuring the DsRed signal using ImageJ software. The spine length, width, and density are 1.53 ± 0.13 μm, 0.44 ± 0.028 μm, and 4.1 ± 0.3 per 10 μm, respectively, in control neurons. *D* and *E*, effects of Rab43N131I on the dendritic expression of α_2B_-AR and M3R. Hippocampal neurons were cultured on 12-well dishes and transfected with 250 ng of α_2B_-AR–GFP (*D*) or M3R–GFP (*E*) together with 1250 ng of DsRed vectors or DsRed–Rab43N131I for 48 h. After fixation, Rab43 and receptor expression were visualized by confocal microscopy. *F*, quantitative data shown in panels *D* and *E*. Dendritic receptor expression was defined as the dendritic area expressing the receptors. *G* and *H*, effects of Rab43N131I on the postsynaptic expression of α_2B_-AR (*G*) and M3R (*H*). *I*, quantitative data shown in panels *G* and *H*. The postsynaptic receptor expression was defined by the ratio of spine over dendritic shaft expression. Bars represent the mean ± SD (n = 25–35 spines in panel *C*, n = 12–20 neurons in panel *F*, and n = 80–95 spines in panel *I* in at least five independent experiments). ∗*p* < 0.05 relative to respective control. In panels *A*, *D*, and *E*, the *scale bars* represent 50 μm, and in panels *B*, *G*, and *H*, the *scale bars* represent 5 μm. α_2_-AR, α_2_-adrenergic receptor; M3R, M3 mAChR.

We next determined the role of Rab43 in the dendritic and postsynaptic transport of α_2_-AR and mAChR, specifically the α_2B_-AR and M3 mAChR (M3R) subtypes, in primary cultures of hippocampal neurons by confocal imaging. We first determined if expression of Rab43N131I could alter the general morphology of neurons. As accessed by the DsRed and GFP signals, expression of DsRed-tagged Rab43N131I had no obvious effect on the general morphology of hippocampal neurons compared with neurons transfected with DsRed vectors (Fig. 2, *A* and *B*). The spine length, width, and density were very much the same in neurons expressing DsRed and Rab43N131I (Fig. 2, *B* and *C*). Consistent with radioligand-binding data, Rab43N131I significantly reduced the dendritic expression of α_2B_-AR by approximately 40%, but had no effect on M3R expression at dendrites (Fig. 2, *D*–*F*). Rab43N131I also significantly attenuated the expression of α_2B_-AR at the dendritic spines by about 35%, whereas M3R expression at the dendritic spines was not affected (Fig. 2, *G*–*I*).

**Figure 3:**
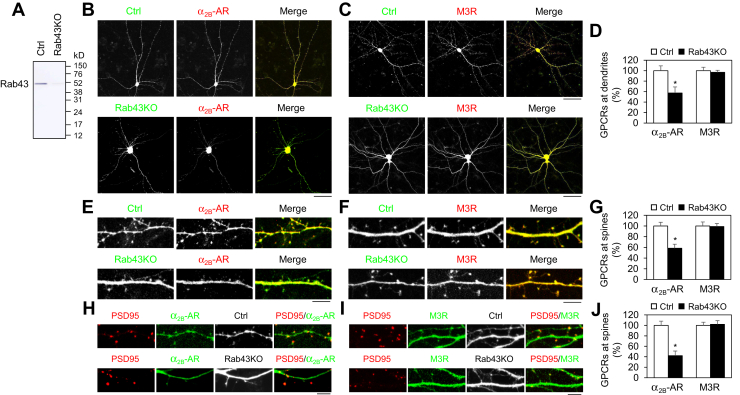
**CRISPR–Cas9–mediated Rab43KO suppresses the dendritic and postsynaptic transport of α**_**2B**_**-AR, but not M3R, in hippocampal neurons.***A*, CRISPR–Cas9–mediated depletion of Rab43. HEK293 cells were transfected with GFP-tagged rat Rab43 together with CRISPR–Cas9 control vectors or KO plasmids targeting mouse Rab43. GFP–Rab43 expression was revealed by Western blotting using GFP antibodies. Similar results were obtained in three experiments. *B* and *C*, effects of Rab43KO on the dendritic expression of α_2B_-AR (*B*) and M3R (*C*). Hippocampal neurons were cultured on 12-well dishes and transfected with 250 ng of α_2B_-AR–CFP (*B*) or M3R–CFP (*C*) together with 500 ng of control or Rab43KO plasmids for 48 h. *D*, quantitative data shown in panel *B* and *C*. *E* and *F*, effects of Rab43KO on the postsynaptic expression of α_2B_-AR (*E*) and M3R (*F*). *G*, quantitative data shown in panels *E* and *F*. *H* and *I*, hippocampal neurons were cultured on 12-well dishes and transfected with α_2B_-AR–YFP (*H*) or M3R–YFP (*I*), RFP-PSD95, and control or Rab43KO plasmids for 48 h. *J*, receptor expression at postsynapses was measured by using PSD95 as a marker. Bars represent the mean ± SD (n = 15–20 neurons in panel *D*, n = 75–82 spines in panel *G*, and n = 62–70 spines in panel *J* in at least five independent experiments). ∗*p* < 0.05 relative to the respective control. In panels *B* and *C*, the *scale bar* represents 50 μm, and in *panelsE*, *F*, *H*, and *I*, the *scale bar* represents 5 μm. α_2_-AR, α_2_-adrenergic receptor; CFP, cyan fluorescent protein; M3R, M3 mAChR; YFP, yellow fluorescent protein.

We then studied the effect of Rab43KO by the CRISPR–Cas9 system on the dendritic and postsynaptic trafficking of α_2B_-AR and M3R in the hippocampal neurons. Owing to the lack to Rab43 antibodies detecting endogenous Rab43 in primary neurons, the efficiency of Rab43KO was tested by using transient expression of GFP-tagged rat Rab43 in human embryonic kidney 293 (HEK293) cells. The expression of rat Rab43 was reduced by more than 90% in cells transfected with Rab43KO plasmids targeting mouse Rab43 as compared with cells transfected with control vectors (Fig. 3*A*), suggesting that CRISPR–Cas9 KO plasmids targeting mouse Rab43 can deplete rat Rab43. In hippocampal neurons, similar to the effects of Rab43N131I, CRISPR–Cas9–mediated Rab43KO markedly inhibited the dendritic presentation of α_2B_-AR, but not M3R (Fig. 3, *B*–*D*). Rab43KO by CRISPR–Cas9 also reduced the expression of α_2B_-AR, but not M3R, at the dendritic spines (Fig. 3, *E*–*G*).

To further confirm the role of Rab43 in the postsynaptic transport, hippocampal neurons were transfected with red fluorescent protein–tagged PSD95, GFP-carrying Rab43KO plasmids, and yellow fluorescent protein–tagged α_2B_-AR or M3R, and receptor expression at postsynapses was measured by using PSD95 as a marker. Postsynaptic expression of α_2B_-AR was markedly reduced, whereas M3R expression remained unaffected in Rab43KO neurons as compared with neurons transfected with control vectors (Fig. 3, *H*–*J*). These data demonstrate distinct functions of Rab43 in the forward transport of α_2B_-AR and M3R to the neuronal dendrites and postsynapses.

### Differential regulation of α_2B_-AR and M3R transport by Rab43 is neuronal cell specific

**Figure 4:**
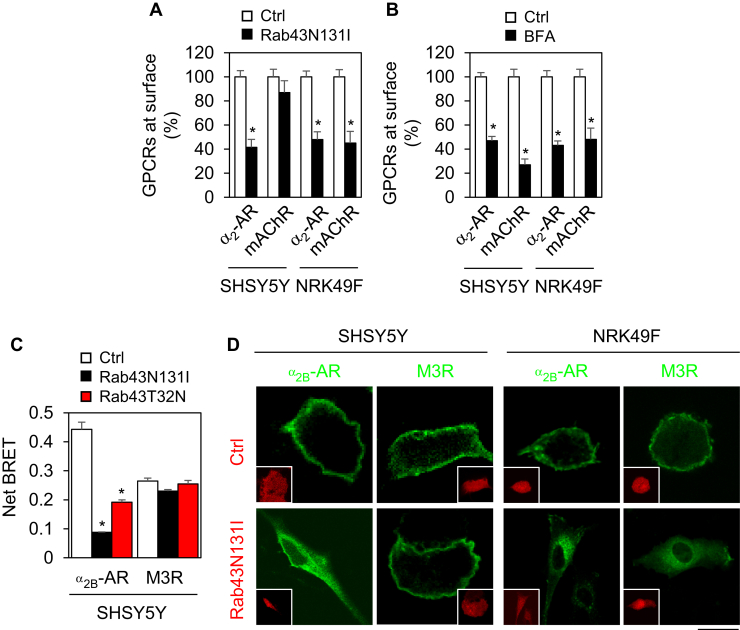
**Differential effects of Rab43N131I on the surface expression of α**_**2**_**-AR and mAChR in neuronal SHSY5Y and non-neuronal NRK49F cells.***A*, effects of Rab43N131I on the surface expression of endogenous α_2_-AR and mAChR. The cells were cultured on 6-well plates and infected with control and Rab43N131I viruses for 48 h. The surface expression of α_2_-AR and mAChR was determined by intact cell ligand binding as described in the legend to Figure 1*B*. In a typical experiment, the mean values of specific ligand binding of α_2_-AR and mAChR were 2336 and 8036 DPM, respectively, in SHSY5Y cells infected with control viruses, and were 2588 and 1831 DPM, respectively, in control NRK49F cells. *B*, BFA treatment attenuates the surface expression of α_2_-AR and mAChR in SHSY5Y and NRK49F cells. The cells were treated with DMSO or BFA at 1 μg/ml for 24 h, and the surface receptor expression was measured by radioligand binding. *C*, effects of Rab43 mutants on the surface expression of α_2B_-AR and M3R in SHSY5Y cells as measured by BRET assays. The cells were cultured on 12-well dishes and transfected with 250 ng of Rluc–α_2B_-AR or Rluc–M3R and 750 ng of venus-kRas together with 1 μg of DsRed–Rab43 or DsRed vectors. *D*, effects of Rab43N131I on the subcellular distribution of α_2B_-AR and M3R. The cells were transfected with GFP–α_2B_-AR or GFP–M3R together with DsRed vectors or DsRed–Rab43N131I. The subcellular localization of the receptors was revealed by confocal microscopy. *Inserts* show the expression of DsRed or DsRed–Rab43. *Bars* represent the mean ± SD (n = 3). ∗*p* < 0.05 relative to respective control. The *scale bar* represents 10 μm. α_2_-AR, α_2_-adrenergic receptor; BRET, bioluminescence resonance energy transfer; M3R, M3 mAChR; mAChR, muscarinic acetylcholine receptor.

We next compared the effects of Rab43 on the surface transport of α_2_-AR and mAChR in human-derived neuroblastoma SHSY5Y cells and rat renal tubular epithelial NRK49F cells. Similar to the results observed from primary cultures of neurons, Rab43N131I expression inhibited the total surface expression of endogenous α_2_-AR in both SHSY5Y and NRK49F cells by greater than 50%, whereas Rab43N131I only attenuated the surface number of endogenous mAChR in NRK49F cells, but not in SHSY5Y cells, as measured by radioligand binding (Fig. 4*A*). In contrast, BFA treatment significantly suppressed the surface expression of α_2_-AR and mAChR in both cell types (Fig. 4*B*).

In bioluminescence resonance energy transfer (BRET) assays to measure receptor surface expression, Rluc-tagged α_2B_-AR or M3R was expressed together with venus-tagged kRas and Rab43 mutants. In addition to Rab43N131I, inactive GDP-bound Rab43T32N mutant was also used. The net BRET signal between α_2B_-AR and kRas was markedly inhibited by both Rab43 mutants, whereas the BRET signal between M3R and kRas was not affected by the Rab43 mutants in SHSY5Y cells (Fig. 4*C*).

Confocal microscopy showed that, as expected, both α_2B_-AR and M3R were robustly expressed at the cell surface in SHSY5Y and NRK49F cells transfected with control vectors. In Rab43N131I-expressing cells, α_2B_-AR was unable to transport to the cell surface and arrested in the perinuclear regions in both cell types, whereas M3R retained its ability to sufficiently move to the cell surface in SHSY5Y cells, but not in NRK49F cells (Fig. 4*D*).

**Figure 5:**
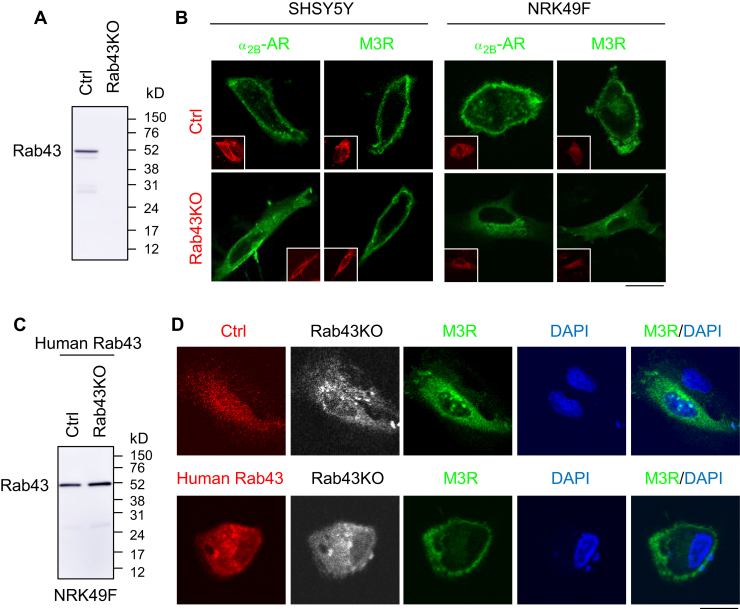
**Differential effects of CRISPR–Cas9–mediated Rab43KO on the surface expression of α**_**2**_**-AR and mAChR in SHSY5Y and NRK49F cells.***A*, CRISPR–Cas9–mediated depletion of Rab43. HEK293 cells were transfected with GFP-tagged human Rab43 together with CRISPR–Cas9 control vectors or KO plasmids targeting human Rab43 for 48 h. GFP–Rab43 expression was revealed by Western blotting using GFP antibodies. *B*, effects of Rab43KO on the subcellular distribution of α_2B_-AR and M3R. The cells were transfected with DsRed-tagged α_2B_-AR or M3R together with CRISPR–Cas9 control vectors or KO plasmids targeting human Rab43 (SHSY5Y cells) or mouse Rab43 (NRK49F cells). Inserts show the expression of the control and Rab43KO plasmids carrying GFP. *C*, human Rab43 expression. NRK49F cells were transfected with DsRed-tagged human Rab43 together with CRISPR–Cas9 control vectors or KO plasmids targeting mouse Rab43. *D*, rescue of the surface expression of M3R by human Rab43 in cells expressing KO plasmids targeting mouse Rab43. NRK49F cells were transfected with GFP-carrying CRISPR–Cas9 KO plasmids targeting mouse Rab43, M3R–CFP, and DsRed vectors or DsRed-tagged human Rab43. In each panel, similar results were obtained in at least three individual experiments. The *scale bars* represent 10 μm. α_2_-AR, α_2_-adrenergic receptor; CFP, cyan fluorescent protein; M3R, M3 mAChR; mAChR, muscarinic acetylcholine receptor.

We then determined the effect of CRISPR–Cas9–mediated Rab43KO on the subcellular localization of α_2B_-AR and M3R. Rab43KO plasmids targeting human Rab43 were tested using GFP-tagged human Rab43 in HEK293 cells (Fig. 5*A*). Similar to Rab43N131I, Rab43KO arrested α_2B_-AR in the perinuclear regions in both SHSY5Y and NRK49F cells, whereas M3R was arrested only in NRK49F cells, but not in SHSY5Y cells (Fig. 5*B*).

We then sought to determine if single-guide RNA (sgRNA)-resistant Rab43 plasmids could rescue the effect of CRISPR–Cas9–mediated Rab43KO on the surface expression of M3R. For this purpose, human Rab43 plasmids were transfected together with mouse Rab43KO plasmids in NRK49F cells. Human Rab43 expression was not affected by mouse Rab43KO plasmids (Fig. 5*C*). Expression of human Rab43 effectively rescued the surface expression of cyan fluorescent protein (CFP)-tagged M3R in NRK49F cells expressing mouse Rab43KO plasmids (Fig. 5*D*). These data demonstrate that different actions of Rab43 on the surface transport of mAChR and specific M3R subtype are neuronal cell type specific.

### Rab43 interaction dictates the sorting of α_2B_-AR and M3R in neuronal cells and primary neurons

**Figure 6:**
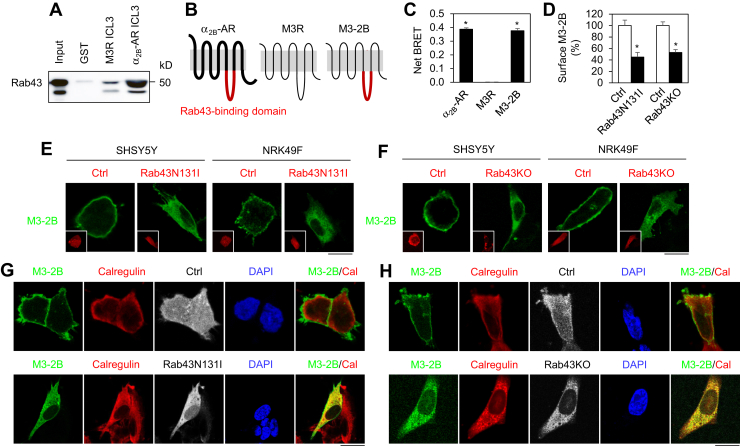
**Rab43N131I and Rab43KO inhibit the cell surface transport of M3-2B from the ER in cells.***A*, differential interaction of the ICL3 of α_2B_-AR and M3R with Rab43 in GST fusion protein pull-down assays. Similar results were obtained in three individual experiments. *B*, a diagram showing the generation of the chimeric receptor M3-2B in which the M3R ICL3 was substituted with the α_2B_-AR ICL3 which binds to Rab43. *C*, Rab43 interaction with α_2B_-AR, M3R, and M3-2B in live cells in BRET assays. *D*, inhibition of the surface expression of M3-2B by Rab43N131I and Rab43KO as measured by radioligand binding assays using [^3^H]-NMS at 20 nM. *E*, effects of Rab43N131I on the subcellular distribution of M3-2B. The cells were transfected with M3-2B–GFP together with DsRed vectors or DsRed–Rab43N131I. *F*, effects of Rab43KO by CRISPR–Cas9 on the subcellular distribution of M3-2B. The cells were transfected with M3-2B–CFP and GFP-carrying CRISPR-Cas9 control vectors or KO plasmids targeting human Rab43 (SHSY5Y cells) or mouse Rab43 (NRK49F cells). *G* and *H*, colocalization of M3-2B with calregulin in cells expressing Rab43N131I (*G*) or Rab43KO (*H*). The cells were transfected with M3-2B–CFP and GFP–Rab43N131I (*G*) or GFP-carrying Rab43KO plasmids (*H*) and then stained with calregulin antibodies. *Inserts* show DsRed (*E*) or GFP (*F*). *Bars* represent the mean ± SD (n = 3). The *scale bars* represent 10 μm. [^3^H]-NMS, [N-methyl-^3^H]-scopolamine methyl chloride; α_2_-AR, α_2_-adrenergic receptor; BRET, bioluminescence resonance energy transfer; CFP, cyan fluorescent protein; ER, endoplasmic reticulum; ICL3, third intracellular loop; M3-2B, M3R mutant containing the α_2B_-AR ICL3; M3R, M3 mAChR.

As our previous studies have shown that α_2B_-AR directly interacts with Rab43 *via* the third intracellular loop (ICL3), to elucidate the molecular mechanisms underlying the selective regulation of α_2B_-AR and M3R trafficking by Rab43 in neurons and neuronal ells, we compared Rab43 interaction with the ICL3 of both receptors in glutathione-*S*-transferase (GST) fusion protein pull-down assays. α_2B_-AR and M3R possess relatively large ICL3, containing 165 and 229 residues, respectively, which were generated as GST fusion proteins. Consistent with our previous data, the α_2B_-AR ICL3 strongly bound Rab43. In contrast, the M3R ICL3 binding to Rab43 was negligible (Fig. 6*A*).

We then generated the chimeric receptor M3-2B in which the M3R ICL3 was substituted with the α_2B_-AR ICL3 (Fig. 6*B*) and measured the possible interactions between Rab43 and individual receptors in live cells in BRET assays. The net BRET signals observed between Rab43 and α_2B_-AR and between Rab43 and M3-2B were similar, but the net BRET signal between Rab43 and M3R was almost undetectable (Fig. 6*C*). These data suggest that the α_2B_-AR ICL3 can confer its interaction with Rab43 to M3R.

We next determined the effect of Rab43 on M3-2B transport in cell lines and primary neurons. In contrast to M3R which is irresponsive to Rab43, the surface expression of M3-2B was inhibited by Rab43N131I and Rab43KO in SHSY5Y cells as measured by intact cell radioligand binding (Fig. 6*D*). Confocal microscopy showed that M3-2B was strongly expressed at the surface which was clearly impeded by Rab43N131I or Rab43KO in both SHSY5Y and NRK49F cells (Fig. 6, *E* and *F*). The intracellularly accumulated M3-2B was extensively colocalized with calregulin, an ER marker (Fig. 6, *G* and *H*). These data suggest that Rab43 is able to control M3-2B transport to the cell surface, specifically its export from the ER.

**Figure 7:**
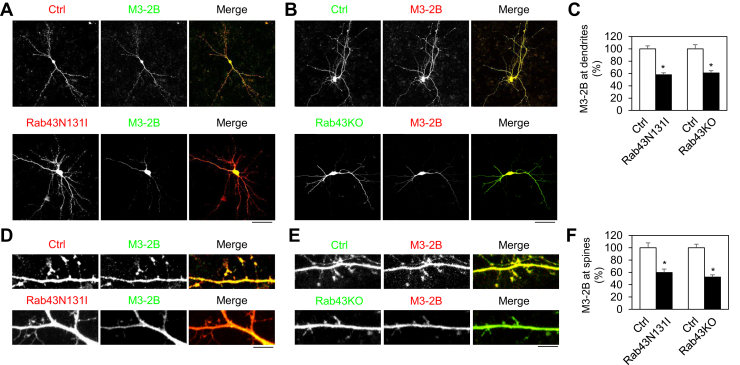
**Rab43N131I and Rab43KO inhibit the dendritic and postsynaptic transport of M3-2B in hippocampal neurons.***A* and *B*, effects of Rab43N131I (*A*) and Rab43KO (*B*) on the dendritic expression of M3-2B. *C*, quantitative data shown in panels *A* and *B*. *D* and *E*, effects of Rab43N131I (*D*) and Rab43KO (*E*) on the postsynaptic expression of M3-2B. *F*, quantitative data shown in panels *D* and *E*. *Bars* represent the mean ± SD (n = 10–15 neurons in panel *C* and n = 65–78 spines in panel *F* in at least three separate experiments). ∗*p* < 0.05 relative to the respective control. In panels *A* and *B*, the *scale bar* represents 50 μm, and in panels *D* and *E*, the *scale bar* represents 5 μm. M3-2B, M3R mutant containing the α_2B_-AR ICL3.

In primary hippocampal neurons, M3-2B was expressed at dendrites and spines. Similar to the results observed in cells, M3-2B expression at dendrites (Fig. 7, *A*–*C*) and spines (Fig. 7, *D*–*F*) was significantly inhibited by Rab43N131I and Rab43KO. Furthermore, the inhibitory effects of Rab43N131I and Rab43KO on the transport of M3-2B were similar (Fig. 7, *C* and *F*). These data demonstrate that the α_2B_-AR ICL3 controls not only M3-2B interaction with Rab43 but also its Rab43-dependent trafficking.

## Discussion

As the first study on the functions of the Rab family GTPases in the anterograde dendritic and synaptic delivery of GPCRs, we have focused on Rab43 which was recently demonstrated to regulate GPCR trafficking in the early secretory pathway in cell (31). The most important finding presented in this article is that, through inhibiting the function of endogenous Rab43 by expression of dominant-negative mutants and CRISPR–Cas9–mediated KO, we have identified Rab43 as an important regulator in the anterograde trafficking of some but not all GPCRs in neurons.

By using radioligand binding of intact live neurons to quantify the exact surface receptor number, we have demonstrated that Rab43 selectively regulates the total surface expression of endogenous α_2_-AR, but not mAChR, in primary neurons. By confocal microscopy to directly visualize the localization of fluorescence-tagged individual receptor subtypes, we have shown that the dendritic and postsynaptic expression of α_2B_-AR, but not M3R, depends on the normal function of Rab43. Such selective actions of Rab43 toward different GPCRs are also reflected by receptor-mediated signaling measured by ERK1/2 activation. These data provide direct evidence indicating novel functions for Rab43 in mediating GPCR transport to dendrites and synapses, as well as their sorting from one another in neurons. However, we cannot exclude the possibility that Rab43 may regulate the trafficking of other signaling molecules involved in receptor-mediated signal transduction pathways, which may also contribute to the observed abnormal signaling, and the role of Rab43 in the trafficking of other α_2_-AR and mAChR subtypes remains unknown.

Similar to the results observed in primary neurons, Rab43 affects the surface expression of endogenous α_2_-AR and specific α_2B_-AR subtype, but not endogenous mAChR and M3R subtype, in neuronal SHSY5Y cells. In contrast, the surface transport of both receptors requires Rab43 in non-neuronal NRK49F cells. These data suggest that the sorting function of Rab43 in the trafficking of distinct GPCRs along the biosynthetic pathways is neuronal cell specific. These data also imply that trafficking itineraries of a GPCR (*e.g.*, M3R) may differ in neurons/neuronal cells and non-neuronal cells.

Although GPCRs share a common structural topology, how they are sorted into distinct transport pathways is poorly understood. Our previous studies suggest that Rab43 interaction may separate GPCRs from non-GPCR plasma membrane proteins (31). Our results presented here have demonstrated that the M3R chimera containing the Rab43-binding domain identified in α_2B_-AR interacts with Rab43 and its dendritic and postsynaptic transport in primary neurons and the surface transport in neuronal cells depend on Rab43. These data indicate that the Rab43-binding domain is able to effectively convert GPCR transport from a Rab43-independent pathway into a Rab43-dependent pathway and that the sorting function of Rab43 is mediated through its direct interaction with the receptors.

An important implication of this study is that different GPCRs may use distinct pathways (*e.g.*, Rab43-dependent and Rab43-independent pathways) to transport from the cell body to dendrites and from the dendritic shaft to spines. It is possible that M3R transport to dendrites is mediated through lateral diffusion as suggested for most GPCRs (5), which is independent of Rab43, whereas α_2B_-AR uses secretory vesicles to move forward to dendrites as suggested for serotonin 1B receptor (5HT1B) (5), which is dependent on Rab43. It is interesting to note that, in addition to exist in the soma, the ER is distributed throughout the cytoplasm in dendrites, the Golgi forms discrete structures called “Golgi outposts” in the longest dendrite, and coat protein complex II (COPII) vesicles that exclusively mediate the ER export of newly synthesized molecules, including GPCRs (34), are formed in distal dendritic branches (35). The dendritic ER, Golgi outposts, and COPII vesicles all participate in the local delivery of plasma membrane receptors (35, 36, 37, 38, 39). We have demonstrated that BFA treatment strongly inhibits the surface expression of both α_2_-AR and mAChR and that Rab43 inhibition arrests the receptors in the ER. These data suggest that the integrity of the Golgi structure plays a crucial role in GPCR export trafficking and that Rab43 controls GPCR trafficking and sorting at the ER level. However, the exact functions of the ER, Golgi compartments, and transport vesicles in the cell body and dendrites in the biosynthesis and sorting of these receptors remain unknown, and the details of these Rab43-dependent and Rab43-independent transport pathways and secretory vesicles carrying Rab43 need further investigation.

The players involved in GPCR targeting in neurons have just begun to be revealed. Recent studies have identified several highly conserved motifs embedded within neuronal GPCRs, which dictate receptor delivery to the surface through regulating receptor correct folding and exit from the ER or the Golgi (6, 7, 40, 41, 42, 43, 44, 45, 46). A number of receptor-interacting proteins have also been identified to control GPCR delivery in neurons, such as Yif1B, in the dendritic targeting of serotonin-1A receptor, prenylated Rab acceptor 1 domain family member 2 in γ-aminobutyric acid B receptor export from the ER, and GGAs (Golgi-associated, γ-adaptin homologous, ADP-ribosylation factor-interacting proteins) in α_2_-AR transport from the Golgi (8, 9, 10, 11, 46, 47, 48). These data, together with our present study, indicate that different GPCRs may use distinct mechanisms, such as interaction with relatively specific regulatory proteins, to achieve their sorting and trafficking to dendrites and synapses.

It is worth pointing out that the separation of GPCRs from one another and from non-GPCR plasma membrane proteins into distinct biosynthetic pathways is a highly regulated, complicated process which is under control by many factors. Our data showing that Rab43 interaction with GPCRs dictates their sorting in neurons and neuronal cells but not in non-neuronal cells suggest that GPCR sorting may be mediated through distinct molecular mechanisms in different cell types. Rab43 interaction with the cargoes it regulates represents only one of these mechanisms by which nascent GPCRs are sorted into Rab43-dependent and Rab43-independent routes that deliver the receptors to the functional destinations in neurons and neuronal cells.

Abnormal GPCR trafficking and signaling are directly linked to pathogenesis of a number of neurologic disorders, for which GPCRs are direct primary therapeutic targets (49, 50). Mutations of Rab GTPases and alterations in Rab-mediated membrane trafficking are also associated with the progression of neurodegenerative diseases, including Alzheimer's, Parkinson's, and Huntington's diseases, and enhanced expression of Rab GTPases may ameliorate neurodegenerative phenotypes (51, 52, 53, 54). In Parkinson’s disease model, overexpression of Rab1, which specifically regulates the ER-to-Golgi transport of newly synthesized proteins including GPCRs (55, 56), is able to successfully rescue the dopaminergic neuron loss caused by α-synuclein accumulation, which inhibits the ER–Golgi trafficking (57). Rab8, a ubiquitously expressed Rab involved in the post-Golgi traffic of GPCRs (58, 59), regulates polarized neurite growth, postsynaptic trafficking of α-amino-3-hydroxy-5-methyl-4-isoxazolepropionate-type glutamatergic receptors (60, 61), and α-synuclein aggregation (26, 62). As such, further studies are needed to define the functional roles of Rab43-mediated GPCR trafficking and sorting in the pathology of neurological diseases. These studies may provide intervention strategies *via* targeting the trafficking processes of GPCRs in the treatment of neurologic diseases involving abnormal function of GPCRs.

In summary, this is the first report of the Rab GTPase family in regulating the dendritic and postsynaptic delivery of GPCRs. Our results have identified novel, neuron-specific functions for Rab43 in the targeting and sorting of GPCRs through direct interaction and suggested distinct yet identified routes for the forward movement of different GPCRs. Overall, this study provides important insights into regulatory mechanisms of maturation processing, trafficking, and sorting of the GPCR members, which are poorly elucidated in sophisticated neurons.

## Experimental procedures

### Materials

Antibodies against GFP, human Rab43, calregulin, phospho-ERK1/2, and β-actin were purchased from Santa Cruz Biotechnology. Antibodies against ERK1/2 were from Cell Signaling Technology. BFA, UK14304, and Oxo-M were obtained from Sigma-Aldrich. The radioligands [^3^H]-RX821002 (50 Ci/mmol) and [N-methyl-^3^H]-scopolamine methyl chloride ([^3^H]-NMS, 80 Ci/mmol) were from PerkinElmer Life Sciences. All other materials were obtained as described elsewhere (63).

### Plasmids and constructions

α_2B_-AR tagged with GFP or CFP at the C-terminus was generated as described previously (42, 64). Similar strategies were used to generate GFP- and CFP-tagged M3R in pEGFP-N1 and pECFP-N1 vectors, respectively. To construct the chimera M3-2B in which the M3R ICL3 (T258-E486) was substituted with the α_2B_-AR ICL3 (K205-E369), the α_2B_-AR ICL3 fragment was amplified by PCR using primers (forward primer, 5’-TTATGACTATTTTATACTGGAGGATCTATAAGGAAAAACGCAGCCACTGCAGA-3’; and reverse primer, 5’-TGAGGGTCTGGGCCGCTTTCTTCTCCCGGCTCAGCTGTGT-3’), and the PCR products were then used to replace the M3R ICL3 by using the QuikChange site-directed mutagenesis kit (Agilent Technologies). Human Rab43 tagged with DsRed or GFP at the N-terminus was generated as described previously (31). Rat Rab43 tagged with GFP at the N-terminus was obtained from GenScript. PSD95–pTagRFP was a gift from Johannes Hall (Addgene #52671). The GST fusion protein construct coding the α_2B_-AR ICL3 was generated as described previously (42, 64). The GST fusion protein construct coding the M3R ICL3 was generated using pGEX-4T-1 vectors at BamH1 and EcoR1 restriction sites and primers (forward primer, 5’-ATGCGGATCCACTGAAAAGCGTACCAAAGAGC-3’; and reverse primer, 5’-ATGCGAATTCCTACTCCTTGACCAGGGACATCC-3’). Rab43N131I and Rab43T32N were generated using the QuikChange site-directed mutagenesis kit. All constructs used in the present study were verified by nucleotide sequence analysis.

### Preparation, lentiviral infection, transient transfection, and drug treatment of primary neurons

Primary cultures of cortical and hippocampal neurons were prepared from embryonic day 18 Sprague-Dawley rat pups (Charles River Laboratories) and grown on glass coverslips precoated with poly-L-lysine in Neurobasal medium supplemented with B27 and L-glutamine. Lentiviruses expressing Rab43N131I were prepared as described (65). To quantify the surface receptor expression by radioligand binding assays, cortical neurons at 12 days *in vitro* were infected with control or Rab43N131I lentiviruses for 2 days. In BFA treatment experiments, cortical neurons at 14 days *in vitro* were incubated with BFA at a concentration of 1 μg/ml for 24 h. To visualize receptor expression at dendrites and postsynapses by confocal microscopy, hippocampal neurons were transfected using Lipofectamine 2000 reagent (Thermo Fisher Scientific) as described previously (11, 66). The use and care of animals used in this study follows the guidelines of the Augusta University Institutional Animal Care and Use Committee. The preparation of primary neurons from timed-pregnant rats was approved by the Augusta University Institutional Animal Care and Use Committee.

### Cell culture and transient transfection

HEK293 and NRK49F cells were cultured in Dulbecco's modified Eagle's medium (DMEM) with 10% fetal bovine serum. SHSY5Y cells were cultured in F12/Minimum essential medium (V/V = 1:1) with 10% fetal bovine serum. Transient transfection of cells was carried out using Lipofectamine 2000 or 3000 reagent as described previously (55).

### CRISPR–Cas9–mediated Rab43KO

The CRISPR–Cas9 Rab43KO plasmids targeting mouse or human Rab43, as well as control plasmids, were purchased from Santa Cruz Biotechnology. The Rab43KO plasmid consists of a pool of three plasmids, each encoding the Cas9 nuclease and a target-specific 20-nt sgRNA. Three sgRNA sequences in mouse Rab43 are TGCCATCGAGACCTCCGCAA, AGTCAGACCTTGCCGATTTC, and CTCACATCCTCGATCCAGTG, which correspond to the sequences TGCCATCGAGACCTCTGCCA, AGTCAGACCTTGCTGATCTC, and CACTGGATTGAGGATGTGAG in rat Rab43. The efficiency of Rab43KO plasmids was tested by using exogenously transfected GFP–Rab43 in HEK293 cells. In brief, HEK293 cells were cultured on 6-well dishes and transfected with 0.5 μg of Rab43KO plasmids. After 6 h, the cells were split at a ratio of 1:2 and cultured overnight. The cells were then transfected with 1.5 μg of Rab43KO plasmids together with 0.5 μg of GFP–Rab43 for additional 24 h. The expression of GFP–Rab43 in total cell lysates was measured by Western blotting using GFP antibodies. To determine the effect of Rab43KO on the subcellular localization of receptors, hippocampal neurons or cells were cultured on 12-well dishes and transfected with Rab43KO plasmids plus individual receptors (0.5 μg each) for 30 h before imaging. In rescue experiments, NRK49F cells were transfected with DsRed-tagged human Rab43, Rab43KO plasmids targeting mouse Rab43 and M3R–CFP (0.5 μg each) for 30 h. As Rab43KO plasmids carry GFP, transfected neurons or cells were defined by the GFP signal.

### Radioligand binding of intact live neurons and cells

The surface expression of endogenous α_2_-AR and mAChR in live neurons and cells was quantified by radioligand binding using [^3^H]-RX821002, which binds all three α_2_-AR subtypes and [^3^H]-NMS, which binds all five mAChR subtypes as described previously (56, 67, 68). In brief, for measurement of endogenous α_2_-AR expression, cortical neurons were cultured in 6-well plates and incubated with 500 μl of Neurobasal medium containing [^3^H]-RX821002 at a concentration of 2 nM for 90 min at room temperature. Nonspecific binding was determined in the presence of rauwolscine at 10 μM. For measurement of endogenous mAChR expression, cortical neurons were incubated with Neurobasal medium plus [^3^H]-NMS at 2 nM for 2 h at 4 °C, and nonspecific binding was determined in the presence of atropine at 20 μM. For measurement of the surface expression of endogenous α_2_-AR and mAChR in cells, cells cultured on 6-well dishes were transfected for 48 h and then incubated with DMEM containing the radioligands at 2 nM. For measurement of the cell surface expression of transiently transfected receptors, cells were incubated with the radioligands at 20 nM. The neurons or cells were then washed twice with 1 ml of PBS, and the surface-bound ligands were extracted by treatment with 500 μl of 1 M NaOH for 2 h. The radioactivity was counted by liquid scintillation spectrometry in 4 ml of Ecoscint A scintillation solution (National Diagnostics, Inc). All radioligand binding assays were performed in duplicate.

### Measurement of ERK1/2 activation

Primary cortical neurons were cultured on 6-well dishes and infected with control or Rab43N131I lentiviruses for 48 h. After starvation for 48 h, the neurons were incubated with Neurobasal medium containing UK14304 at 1 μM or Oxo-M at 10 μM for 5 min. The neurons were then washed twice with cold PBS and solubilized by the addition of 100 μl of the SDS gel-loading buffer. ERK1/2 activation was determined by measuring their phosphorylation by Western blotting as described previously (55, 63).

### Confocal microscopy

For image acquisition and quantification of receptor expression at dendrites and postsynapses, hippocampal neurons were fixed with 4% paraformaldehyde and 4% sucrose for 15 min and washed with PBS for three times. Images were captured with a 40× objective on a Zeiss LSM780 confocal microscope. Confocal images were analyzed and quantified with the ImageJ software (the National Institutes of Health). Dendritic receptor expression was measured as the dendritic area expressing individual receptors as described previously (58). To measure receptor expression at postsynapses, spines of secondary dendrites and adjacent dendrite shaft regions were defined under the DsRed or GFP channel, and postsynaptic receptor expression was measured by the ratio of spine over dendritic shaft expression.

For analysis of receptor localization in SHSY5Y and NRK49F cells, the cells were grown on coverslips precoated with poly-L-lysine on 6-well dishes and transfected with 50 ng of receptors together with 150 ng of Rab43N131I or Rab43KO plasmids for 48 h. To study receptor colocalization with calregulin, the cells were fixed and permeabilized with PBS containing 0.2% Triton X-100 for 5 min. After blocking with 5% normal donkey serum for 1 h, the cells were sequentially stained with primary antibodies against calregulin (1:100 dilution) for 1 h and fluorophore-conjugated secondary antibodies (1:2000 dilution) for 1 h. Images were captured with a 63× objective on a Zeiss LSM780 confocal microscope as described previously (63).

### GST fusion protein pull-down assays

GST fusion protein pull-down assays were carried out by using the MagneGST Pull-Down System as described previously (48, 64). Briefly, GST and GST fusion proteins were expressed in BL-21 bacteria and purified using glutathione beads. The purity of fusion proteins was analyzed by Coomassie brilliant blue staining after SDS-PAGE before experiments. GST fusion proteins tethered to the glutathione beads were either used immediately or stored at 4 °C for no longer than 2 days. GST fusion proteins were incubated with total lysates prepared from HEK293 cells expressing GFP–Rab43 in 500 μl of the binding buffer containing 20-mM Tris HCl, pH 7.5, 140-mM NaCl, 1% Nonidet P-40, 0.5% bovine serum albumin, and 10% glycerol overnight at 4 °C. The resin was washed four times with 1 ml of the binding buffer. The bound proteins were solubilized in the SDS gel-loading buffer and detected by immunoblotting using GFP antibodies.

### BRET assays

The live cell–based BRET assays were used to measure the cell surface expression of GPCRs, as well as Rab43 interaction with GPCRs, in SYSH5Y cells as described previously (64, 69). To measure the surface expression of GPCRs, the cells were cultured on 12-well dishes and transfected with 250 ng of Rluc-tagged receptors and 750 ng of venus-tagged kRas or pcDNA3.1 together with 1 μg of DsRed-tagged Rab43N131I or DsRed vectors with Lipofectamine 3000. To measure possible interaction between Rab43 and individual GPCRs, the cells were transfected with 250 ng of Rluc-tagged Rab43 and 750 ng of venus-tagged receptors or pcDNA3.1 vector for 12 h. The cells were transferred to 6-well dishes and cultured for additional 36 h. The cells were then harvested and split onto black 96-well plates. After addition of coelenterazine h (5 mM), luminescence was immediately measured using a Mithras LB940 photon-counting plate reader (Berthold Technologies GmbH). Raw BRET signals were calculated by dividing the emission intensity at 520 to 545 nm by the emission intensity at 475 to 495 nm. Net BRET was this ratio minus the same ratio measured from cells expressing only the BRET donor (Rluc).

### Statistical analysis

Differences were evaluated using Student's *t* test, and *p* < 0.05 was considered as statistically significant. Data are expressed as the mean ± SD.

## Data availability

All data presented are available upon request from Guangyu Wu (guwu@augusta.edu).

## Conflict of interest

The authors declare that they have no conflict of interest with the contents of this article.
